# Enhancement of the killing effect of low-temperature plasma on *Streptococcus mutans* by combined treatment with gold nanoparticles

**DOI:** 10.1186/s12951-014-0029-5

**Published:** 2014-08-08

**Authors:** Sang Rye Park, Hyun Wook Lee, Jin Woo Hong, Hae June Lee, Ji Young Kim, Byul bo-ra Choi, Gyoo Cheon Kim, Young Chan Jeon

**Affiliations:** 1 Department of Dental Hygiene, Kyungnam College of Information and Technology, Busan 617-701, Rep. Korea; 2 Department of Electrical Engineering, Pohang University of Science and Technology, Pohang 790-784, Rep. Korea; 3 Department of Korean Internal Medicine, School of Korean Medicine, Pusan National University, Yangsan 626-870, Korea; 4 Department of Electronics Engineering, Pusan National University, Busan 609-735, Rep. Korea; 5 Department of Oral Anatomy, School of Dentistry, Pusan National University, Yangsan 602-739, Rep. Korea; 6 Department of Dental Prosthetics, School of Dentistry, Pusan National University, Yangsan 602-739, Republic of Korea

**Keywords:** Gold nanoparticle, Low-temperature plasma, Streptococcus mutans, Sterilization, Oral care

## Abstract

**Background:**

Recently, non-thermal atmospheric pressure plasma sources have been used for biomedical applications such as sterilization, cancer treatment, blood coagulation, and wound healing. Gold nanoparticles (gNPs) have unique optical properties and are useful for biomedical applications. Although low-temperature plasma has been shown to be effective in killing oral bacteria on agar plates, its bactericidal effect is negligible on the tooth surface. Therefore, we used 30-nm gNPs to enhance the killing effect of low-temperature plasma on human teeth.

**Results:**

We tested the sterilizing effect of low-temperature plasma on *Streptococcus mutans* (*S. mutans*) strains. The survival rate was assessed by bacterial viability stains and colony-forming unit counts. Low-temperature plasma treatment alone was effective in killing *S. mutans* on slide glasses, as shown by the 5-log decrease in viability. However, plasma treatment of bacteria spotted onto tooth surface exhibited a 3-log reduction in viability. After gNPs were added to *S. mutans*, plasma treatment caused a 5-log reduction in viability, while gNPs alone did not show any bactericidal effect. The morphological changes in *S. mutans* caused by plasma treatment were examined by transmission electron microscopy, which showed that plasma treatment only perforated the cell walls, while the combination treatment with plasma and gold nanoparticles caused significant cell rupture, causing loss of intracellular components from many cells.

**Conclusions:**

This study demonstrates that low-temperature plasma treatment is effective in killing *S. mutans* and that its killing effect is further enhanced when used in combination with gNPs.

## Background

Dental caries is a chronic infection of worldwide prevalence, and it represents oral health problems associated with oral bacteria [1]. Although remarkable technical developments have been made in dental treatment, dental caries remains a major oral health problem in most countries [2]. Dental caries commonly occur on the occlusal and proximal surfaces of the tooth, particularly in its pits and fissures. These sites are structurally difficult to approach when treating a decayed tooth. In clinical management, a carious tooth is simply removed by dental handpiece drilling, after which a restorative material is used to fill the empty space. During this process, a greater amount than necessary of healthy tooth tissue is often removed along with the decayed part. Furthermore, if the cavity is filled without completely removing bacteria, the remaining bacteria cause recurrence of dental caries. Therefore, a novel method that strongly inhibits the causative pathogens regardless of their spatial accessibility to hand-held tools and decreases the excessive removal of the healthy parts of the tooth is highly desirable.

Low-temperature atmospheric pressure plasma has been used for biomedical applications such as sterilization, cancer treatment, blood coagulation, and wound healing [3]. Since plasma generates high amounts of reactive oxygen species (ROS) and hydroxyl radicals (•OH), it is highly effective in killing bacteria such as *Escherichia coli*, *Candida albicans*[4],[5], *Pseudomonas aeruginosa*[6], and *Lactobacillus casei*[7]. The bactericidal property of non-thermal plasmas has been recently used against oral pathogens. Effective killing of *Enterococcus faecalis* has been reported using low-frequency air plasma and pulse-modulated He plasma needles [8]. A microwave plasma pencil has been reported to induce a high death rate of *Streptococcus mutans* with H_2_O_2_[9]. However, plasma treatment alone needs a relatively long time for killing *S. mutans*[9],[10].

*S. mutans*, a facultative anaerobic [11] and high acid-producing bacterium [12], has been identified as the principal cause of dental caries. Acidic substances produced by *S. mutans* destroy the dental enamel, leading to severe tooth decay. Plasma treatment has been reported to induce a high death rate of *S. mutans*. However, in those studies, the plasma temperature was too high for application to human tissues. [13],[14]. Being gram-positive, *S. mutans* has a thick cell wall to protect it from the external environment. This thick cell wall makes it more difficult to kill gram-positive than gram-negative bacteria. Furthermore, the presence of gram-positive bacteria in very narrow crevices of a tooth limits their accessibility for various treatments. In this study, gold nanoparticles (gNPs) were used to enhance the killing effect of low-temperature plasma on *S. mutans*. Because of the high electric conductivity of gNPs, we assumed that they could be activated by plasma. Stimulated gNPs would then cause high levels of cellular stress, which could lead to the death of the bacteria. The aim of this study was to enhance the killing effect of low-temperature plasma on *S. mutans* by means of gNPs for dental caries treatment.

## Results

### Enhancement of the killing effect of plasma by using gNPs

**Figure 1:**
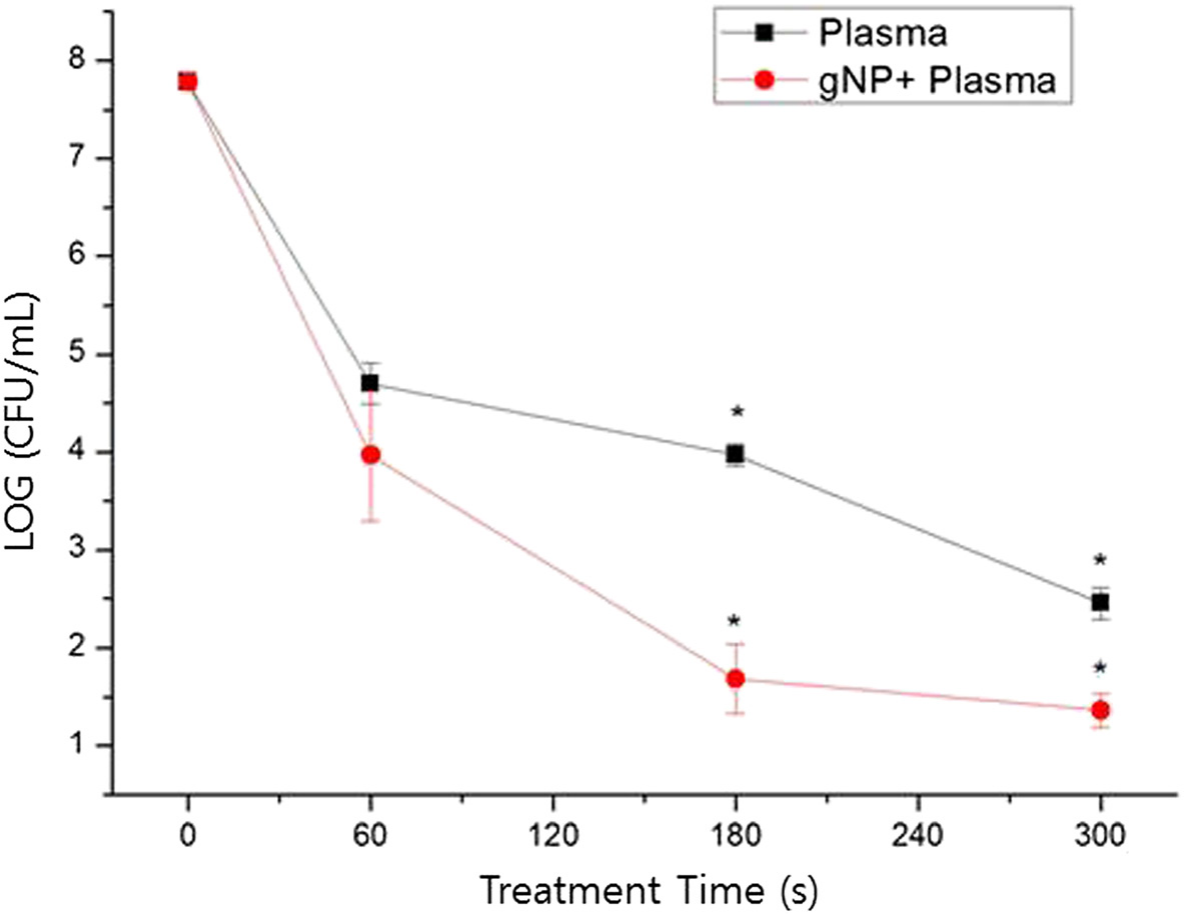
**Enhanced killing effect can be achieved using combined gold nanoparticle (gNP) and low-temperature plasma treatment for*Streptococcus mutans*cultured on a cover glass.** Bacterial viability was assessed by plate counting. The difference between the death rates for plasma-only and plasma plus gNPs was statistically significant (*p* < 0.05).

**Figure 2:**
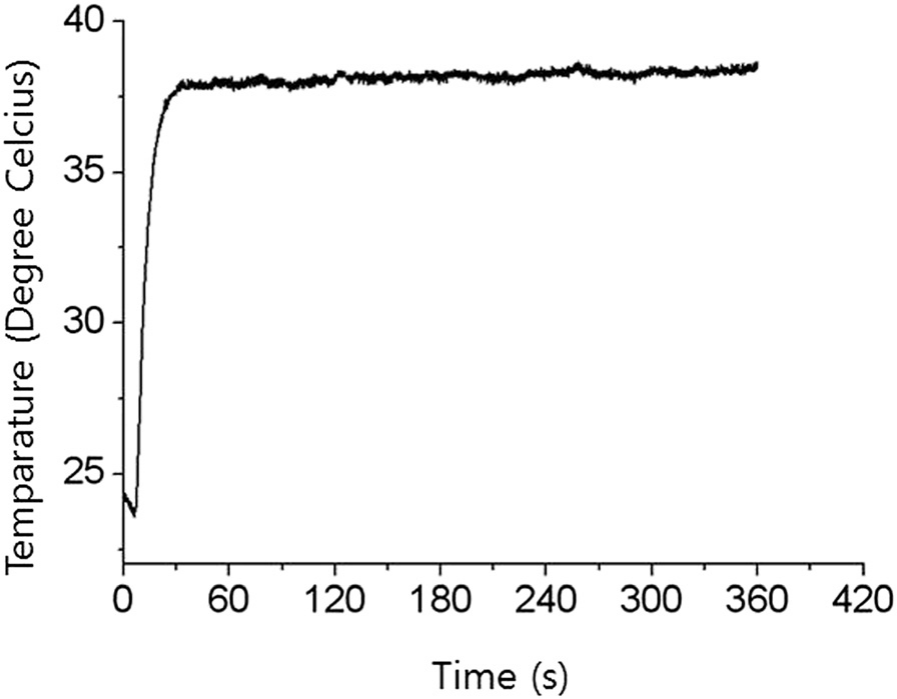
The temperature of the plasma jet was measured 8 mm away from the end of the plasma source with an argon flow rate of 2.5 standard liters per minute.

To investigate the killing effect of plasma, a cover glass was coated with 5 μL (~10^8^ colony-forming units (CFUs)/mL) of a suspension of *S. mutans*. The survival curve showed a 5-log reduction in cell viability by plasma treatment for 300 s. Using gNPs attached to *S. mutans* cells in combination with plasma treatment yielded a 6-log reduction in viability (Figure 1). The killing effect of plasma on *S. mutans* cannot be due to a thermal effect because the plasma temperature did not exceed 37°C for longer than 5 min (Figure 2).

**Figure 3:**
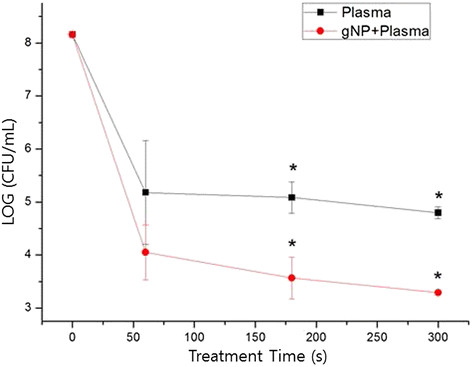
**Enhanced killing effect can be achieved using a combination of gNPs and low-temperature plasma treatment for*S. mutans*on the tooth.** Bacterial viability was typically assessed by plate counting. The difference between the death rate of *S. mutans* by using plasma alone and that by using plasma plus gNP is statistically significant (*p* < 0.05).

The killing effect of plasma on *S. mutans* cells was lower for cells spotted onto the surface of the tooth than for cells on a cover glass. To enhance the bactericidal effect, gNPs were employed. After the addition of gNPs to *S. mutans* for 1 h, plasma treatment significantly decreased the viability of *S. mutans* cells. Plasma-only treatment of *S. mutans* cells for 300 s showed a 3-log reduction, while cells treated with gNPs and plasma exhibited a 5-log decrease (Figure 3). The difference in the killing effect with plasma only and combination treatment with gNPs and plasma was statistically significant (*p* < 0.05).

### Viability staining of *S. mutans* cells

**Figure 4:**
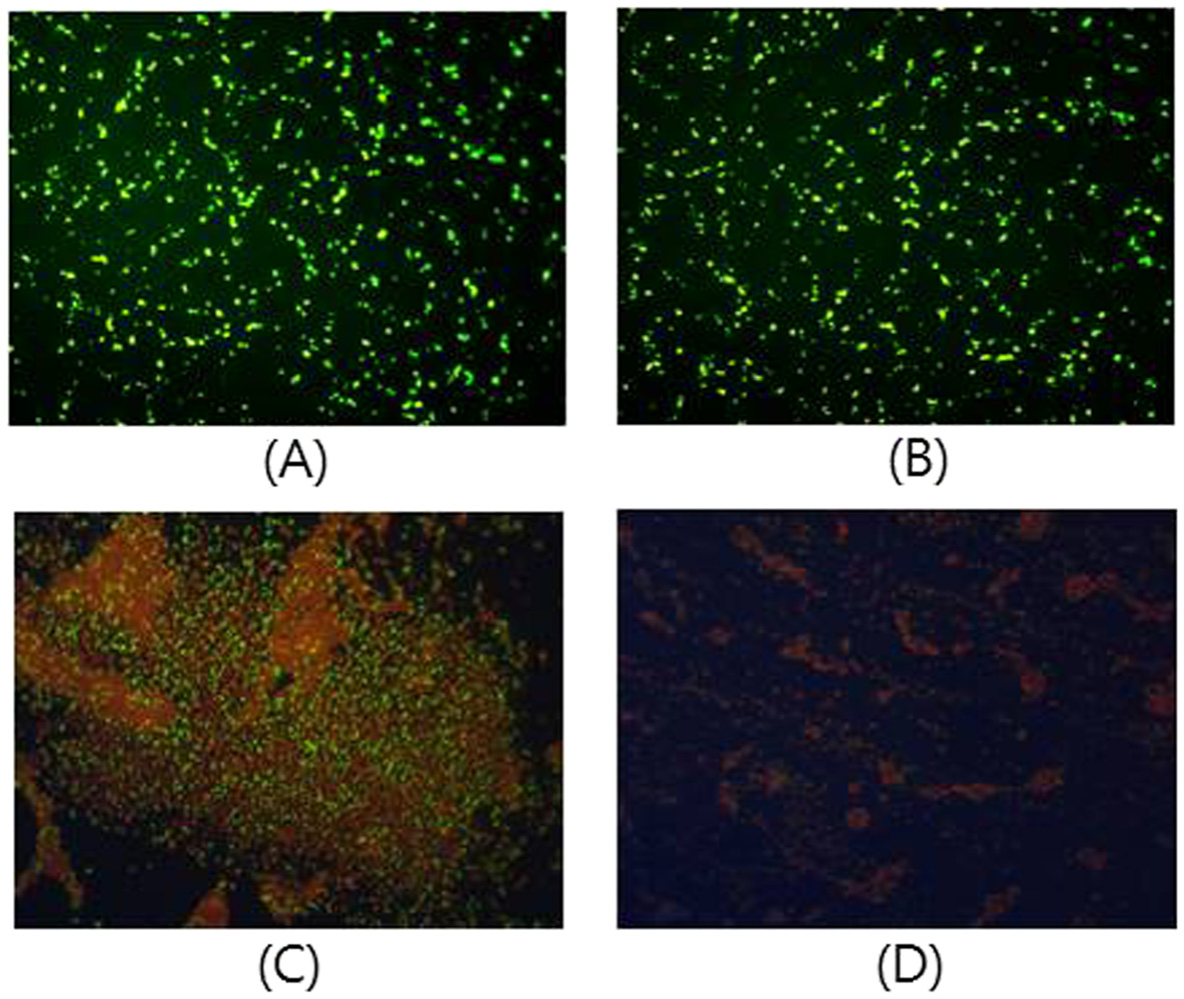
**Viability staining of*S. mutans*cells. (A)** Untreated control cells. **(B)***S. mutans* cells treated with gNPs. **(C)***S. mutans* cells treated with plasma only. **(D)***S. mutans* cells treated with gNPs and plasma. All treatments were applied for 30 s, and cells were stained with SYTO 9 and propidium iodide. Images were observed under a fluorescence microscope (magnification, ×400).

The viability of *S. mutans* was analyzed using fluorescence microscopy. *S. mutans* was stained with SYTO 9® and propidium iodide (PI). SYTO 9® (green fluorescence) can label all living bacteria in a population, whereas PI (red fluorescence) can only penetrate bacteria with damaged membranes, which causes a decrease in SYTO 9® fluorescence intensity. Control cells and bacteria treated only with gNPs showed green fluorescence (Figure 4A and B), while red fluorescence was detected in *S. mutans* cells treated with microwave plasma for 60 s (Figure 4C). The combination treatment of plasma and gNPs showed only a few green fluorescent cells (live), which were detected among mostly red fluorescent cells (dead) (Figure 4D).

**Figure 5:**
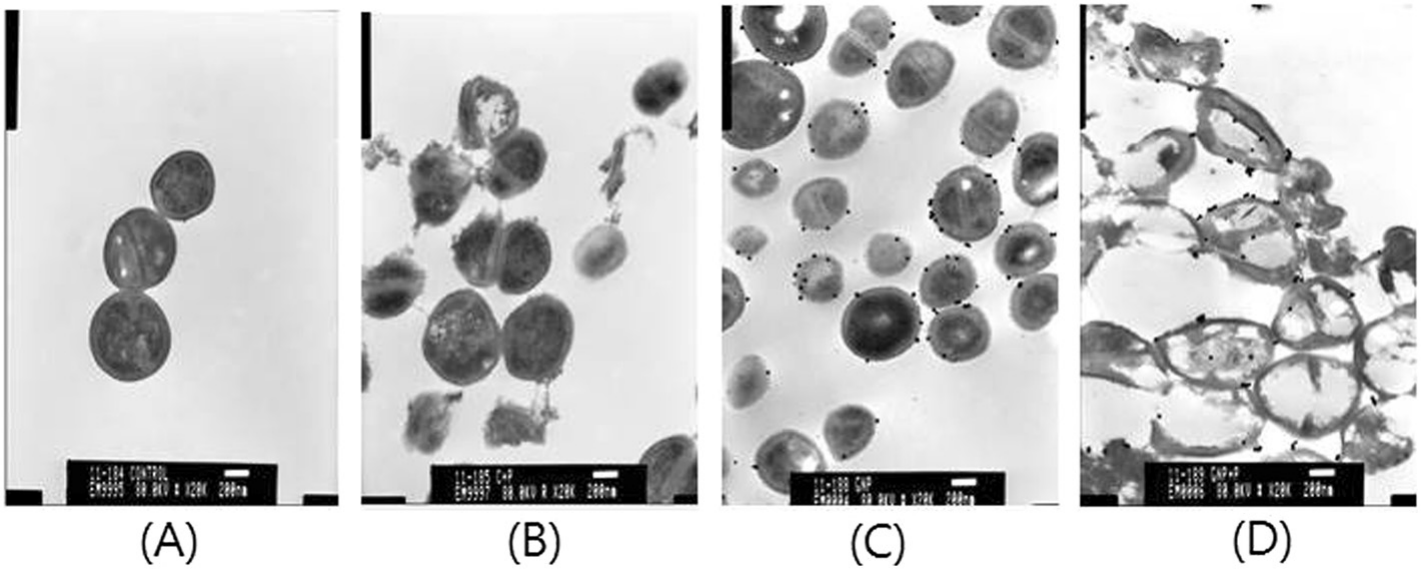
**Transmission electron microscopy images of*S. mutans*treated with low-temperature plasma and gNPs (×20,000). (A)** Control cells without any treatment. **(B)***S. mutans* cells treated with plasma only. **(C)***S. mutans* cells treated with gNPs only. **(D)** Morphological features of *S. mutans* cells after combined treatment with plasma and gNPs.

### Morphological examination of cell damage by transmission electron microscopy

Using transmission electron microscopy (TEM), we analyzed the morphological changes in *S. mutans* cells after plasma treatment. The cell membrane was smooth, and cell division was observed in the control experiment (Figure 5A). After plasma treatment, the cell membrane and cell wall were disrupted, but the cytosol was retained within the cell (Figure 5B). After addition of gNPs, which bind to the cell wall of *S. mutans*, the plasma treatment significantly ruptured the cell walls (Figure 5C) and led to the release of its cytoplasmic components (Figure 5D).

## Discussion

This study demonstrates that gNPs enhance the killing effect of low-temperature plasma on *S. mutans*. Conventionally, oral antiseptic agents have been widely used to reduce the number of oral bacteria. However, their usage for long periods might lead to side effects such as tooth staining, dry mouth, and taste disturbance [15]. Currently, lasers are being used in many fields of dentistry, including caries treatment [16]. A laser beam is an intense, coherent, and highly directional beam of light. These characteristics of lasers are of limited benefit for cavity treatment in a tooth with a curved surface and an irregular shape. For instance, in the case of periapical infections, it is difficult to apply laser beams to the infection site. However, plasma is an ionizing gas, which results in high flexibility for application to various oral structures and for overcoming such spatial limitations. Thus, plasma can easily approach an infection site and effectively kill oral pathogens.

Although many studies have been carried out to determine the mechanism of killing by plasma, the exact mechanism is not yet clearly understood. This might be due to the multitude of plasma-generated components such as ROS, charged particles, and electrostatic and electromagnetic fields. Thus, it is possible that several components work together to produce a synergistic effect rather than a single component contributing to the sterilization. Plasma can produce a large amount of ROS when it passes through air, and in particular, high levels of •OH are generated when plasma reacts with water or tissue fluid. It is well known that •OH effectively kills bacteria. Furthermore, the half-life of plasma-generated ROS is very short, and hence, its retention in the oral cavity is short and less likely to induce harmful effects on tissues. Although the plasma is called low-temperature plasma, plasma gas generates heat, which should not lead to thermal damage to tissues. In other articles reporting the killing effect of plasma, thermal damage could be observed owing to the high temperature of plasma [13],[14]. Considering that temperatures higher than 42.5°C induce pulpal damage, the plasma temperature should be maintained below 40°C. As shown in Figure 2, the temperature of the plasma used in this experiment did not exceed 37°C for longer than 5 min. Thus, plasma-induced thermal damage to the oral tissues was unlikely.

In this study, plasma treatment showed a 5-log reduction in *S. mutans* cells on the cover glass. This significant bactericidal effect decreased to a 3-log reduction when *S. mutans* was grown on the tooth surface. Although a 3-log reduction of *S. mutans* obviously represents a high bactericidal effect, the residual bacteria could cause recurrent dental caries. Accordingly, as a new method to overcome this issue, we used gNPs in combination with low-temperature plasma to achieve a high level of effectiveness and rapid killing of *S. mutans*. In this study, we used 30-nm colloidal shaped gNPs. gNPs are well known for biosafety and uptake into the cell [17]. The shape and size of gNPs can be easily controlled [18], which has led to their widespread application in diagnostics [19], therapeutics, and drug delivery [20]. According to one study, the electric field at the adhesion point between gNPs and membrane was amplified when gNPs attached to the surface of a nuclear membrane were exposed to plasma [21]. In an earlier study by our group, we showed that gNPs stimulated by plasma induced selective cancer cell death in melanoma and oral squamous carcinoma cells [22],[23]. In the current study, gNPs were bound to *S. mutans* cells walls, and no alterations were observed in the *S. mutans* cells. Plasma treatment of *S. mutans* was more effective against gNP-attached cells than against the gNP-free ones. TEM images showed severe cell wall damage with plasma treatment. Furthermore, the combination treatment of gNPs and plasma led to cell wall rupture, such that most intracellular components were released. This finding suggests that the plasma energy, which may have been amplified by gNPs, could induce severe stress to the *S. mutans* cell walls*.* The most likely mechanisms of the synergistic effect of the microwave plasma and gNPs might be enhanced electric field and local heating near the gNPs. High electric conductivity and the nano-size geometry of the gNPs lead to electric field concentration on gNPs [21]-[23]. The enhanced electric field near the gNPs might attract or repel charged particles. This could cause ion bombardment from the plasma [24] and gathering of charged particles inside bacteria, leading to the rupture of the cell wall. Joule heating of the gNPs can be an important factor. An earlier study showed high thermal power dissipation of gold by a radio frequency electric field, and it increased as the size of gNPs decreased [25]. These suggestions can explain the severe damages to the bacteria cell walls.

Considering these data, the use of gNPs in combination with plasma has proven very effective in killing the bacteria hidden in the very small spaces of the tooth. Therefore, this technique can limit the number of bacteria remaining within the tooth structure and markedly decrease the recurrence of dental caries.

The microwave plasma jet has a resonator structure. Most of the microwave power is consumed for sustaining the plasma and is reflected at the open end of the microwave plasma source [26]. Only little microwave power can leak from the microwave plasma source. A low E-field has been found to exist near the open end, and the E-field intensity reduces exponentially as the distance increases [27]. This indicates that the effect of microwave leakage on patients is negligible.

## Conclusions

Low-temperature plasma can be applied to various tooth structures for therapy and preventive dentistry. Furthermore, plasma-stimulated gNPs attached to *S. mutans* significantly destroyed the cells walls, thereby promoting cell death. We suggest that the technique using plasma and gNPs could be a good method for dental caries treatment in dental clinics.

## Methods

### Plasma source

**Figure 6:**
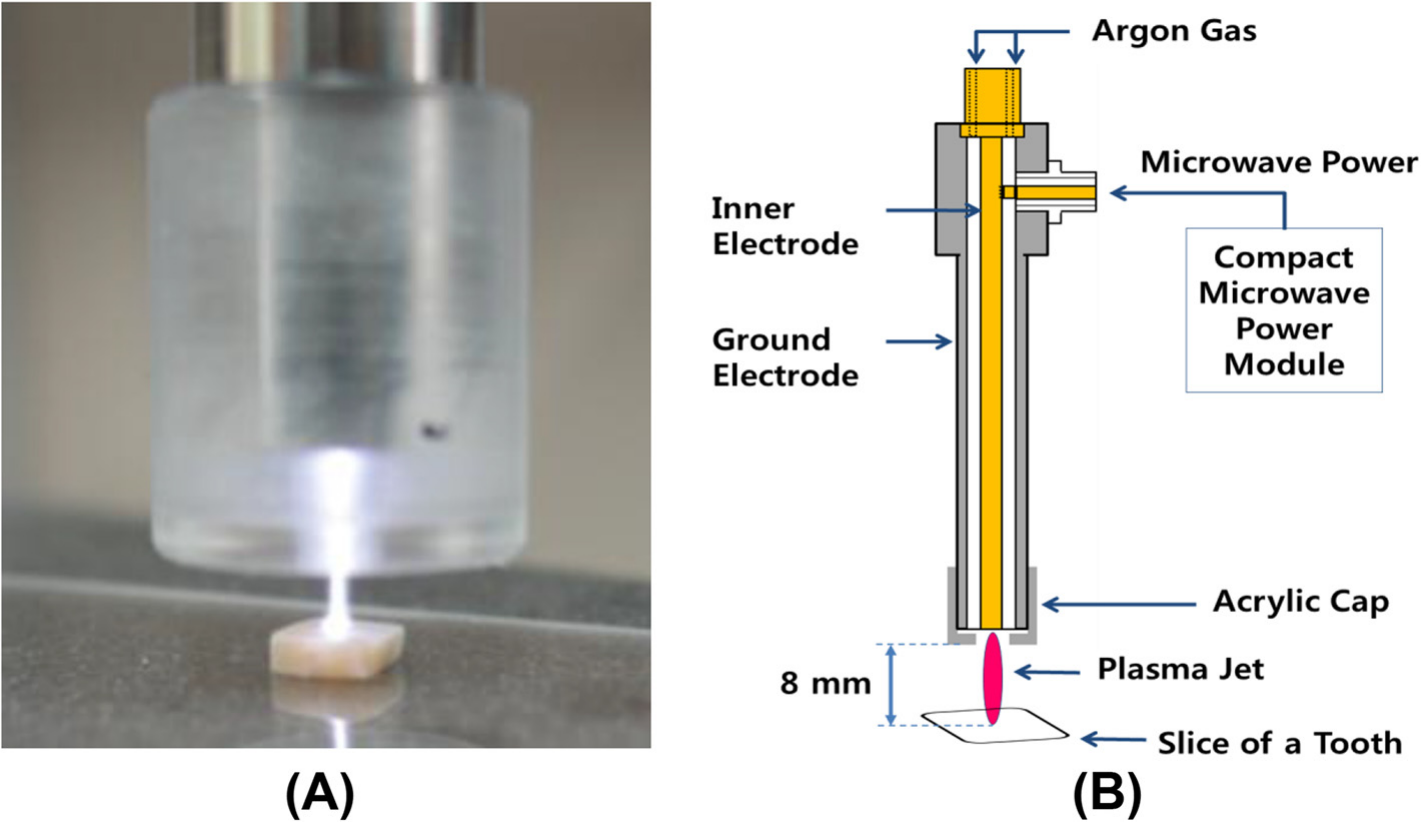
**Microwave plasma device and plasma jet. (A)** Microwave plasma source and **(B)** schematic diagram of the plasma device and the experimental set up.

Microwave driven–atmospheric pressure plasma was employed for killing *S. mutans*. The plasma source was based on the coaxial transmission line resonator [26], and it generated a low-temperature plasma jet. It was operated by a palm-size power module [28] with a 2.5 W net input power. Argon gas was supplied to the plasma source through a flow meter (KOFLOC, Ar-05). We covered the end of the plasma source with an acrylic cap that had a smaller output area (diameter of output hole, 2 mm) than the plasma source. The stability of the plasma jet at the acrylic cap increased without change in the characteristics of the plasma (Figure 6).

### Temperature measurement

Generally, microwave-driven plasma has high temperatures. However, by using a very low operating power (2.5 W) and adjusting the gas flow, we could obtain low-temperature plasma. Under constant input power, the temperature of the plasma jet was dependent of the gas flow rate. The temperature of the plasma jet was measured using a The temperature of the plasma jet was measured using a thermometer with an optical fiber probe (FTI-10, FISO Technologies). The probe was located 8 mm away from the end of the plasma source, and the plasma jet contacted the probe. The location of the probe coincided with the location of the target tooth in the sterilization experiment. The maximum temperature reached was 41.1°C, and it decreased to 37°C in 5 min (Figure 2). The gas flow rate was set to 2.5 standard liters per minute. The length and diameter of the plasma jet were approximately 8 mm and 2 mm, respectively.

### Tooth slicing and sample preparation

Tooth slices of 2 mm thickness and approximately 3 mm diameter were cut from extracted human molars with a low-speed diamond saw (Struers, Copenhagen, Denmark). The exposed tooth surface was smoothened with Sic abrasive paper with grinding (grit P 120, 800, 1200). Subsequently, the tooth slices were cleaned for 10 min in the ultrasonic cleaner, autoclaved at 121°C for 15 min, and stored in phosphate-buffered saline (PBS). Five microliters of the *S. mutans* solution was spotted onto the tooth surface and allowed to dry out for 15 min at room temperature. The distance between the plasma jet and the tooth surface was 8 mm. The contamination on the tooth surface was plasma-treated for 60 s, 180 s, and 300 s. After plasma treatment, the tooth slices were vortexed for 30 s in 1 mL PBS, and 100 μL of these samples was inoculated onto agar plates in a 10-fold serial dilution. The agar plates were incubated at 37°C for 24 h before the colonies were counted (IRB No. 05-2012-023).

### Treatment of bacteria on cover glass

*S. mutans* on cover glass were treated with non-thermal plasma. All conditions were performed in the same manner as in experimental tooth slices.

### Bacterial strains and culture conditions

The strain of *S. mutans* (KCTC 3065/ATCC 25175) was grown in brain heart infusion (BHI) broth (Fluka, Switzerland). *S. mutans* was cultivated overnight in liquid media incubated at 37°C, and the cells were diluted with PBS to a final concentration of approximately 10^8^ CFUs/mL.

### Preparation of gNPs

Effective binding of *S. mutans* was achieved using 30-nm colloidal gNPs. *S. mutans* cells were cultured in BHI broth medium for 24 h at 37°C and harvested by centrifugation at 4,000 rpm for 5 min at 4°C. The supernatant was discarded, and 10 mM sodium bicarbonate (pH = 8.8) was added to the gNPs to resuspend them. *S. mutans* cells were added to the gNPs and incubated for 1 h at 37°C. Subsequently, they were centrifuged at 4°C for 5 min at 4,000 rpm. The supernatant was discarded, and sodium bicarbonate buffer was used for the final suspension solution.

### Bacterial viability staining

After the plasma treatment, *S. mutans* was stained with the nucleic acid stains SYTO 9® (Invitrogen-Molecular Probes, OR, USA) and PI (Sigma Aldrich, MO, USA) according to the manufacturer’s instructions. Cells were visualized and classified as live or dead by fluorescence microscopy (Axioskop; Zeiss, Germany).

### Transmission electron microscopy

Bacterial samples for TEM imaging were fixed with 2.5% glutaraldehyde for 2 h at 4°C and then incubated with 1% osmium tetroxide for 1 h at 4°C. After the cells were rinsed, dehydrated in ethanol, and infiltrated with propylene oxide, they were embedded in Epon. Ultrathin sections were stained with uranyl acetate and lead citrate and observed using TEM at Electron microscopy Facility, Department of Ophthalmology, Pusan National University Hospital (JEM 1200EX-II; JEOL, Japan).

### Statistical analysis

Statistical analysis was performed using SPSS (SPSS version 18 for Windows, SPSS Inc., USA). The logarithmic values of each bacterial plate count were calculated and analyzed using the Student’s *t*-test. A *p*-value less than 0.05 was considered statistically significant.

## Abbreviations

BHI: Brain heart infusion

CFU: Colony-forming unit

gNP: Gold nanoparticle

PBS: Phosphate-buffered saline

PI: Propidium iodide

TEM: Transmission electron microscopy

## Competing interests

The authors declare that they have no competing interests.

## Authors' contributions

HJL analyzed the data, and YCJ contributed in collecting tooth samples. SRP, HWL, and BRC carried out the laboratory experiments and analyzed the data. GCK provided technical input and devised the project idea. SRP and GCK wrote the manuscript. JYK contributed to interpretation of data. All authors read and approved the final manuscript.
